# Family Planning in Fertile-Age Patients With Multiple Sclerosis (MS) (ConPlanEM Study): Delphi Consensus Statements

**DOI:** 10.7759/cureus.44056

**Published:** 2023-08-24

**Authors:** Celia Oreja-Guevara, Mar Tintoré, Virginia Meca, José María Prieto, José Meca, Mar Mendibe, Alfredo Rodríguez-Antigüedad

**Affiliations:** 1 Neurology, Hospital Clínico San Carlos, Madrid, ESP; 2 Neurology, Multiple Sclerosis Center of Catalonia (Cemcat) Vall d'Hebrón University Hospital, Barcelona, ESP; 3 Neurology, Princess University Hospital, Madrid, ESP; 4 Neurology, University Clinical Hospital of Santiago de Compostela, Madrid, ESP; 5 Neurology, Multiple Sclerosis CSUR and Clinical Neuroimmunology Unit, Virgen de la Arrixaca Clinical University Hospital, Cartagena, ESP; 6 Neurology, Neuroimmunology Group, Biocruces Bizkaia Research Institute, Cruces University Hospital, Bizkaia, ESP; 7 Neurology, Cruces University Hospital, Bizkaia, ESP

**Keywords:** delivery, intrapartum, postpartum, disease-modifying treatment, pregnancy, multiple sclerosis

## Abstract

Family planning is essential for establishing Multiple Sclerosis (MS) prognosis, treatment decision, and disease monitoring. We aimed to generate an expert consensus addressing recommendations for family planning in MS patients of childbearing age.

Initially, a committee comprising seven neurologists, experts in the MS field, identified the topics to be addressed. Then, the committee elaborated on different evidence-based preliminary statements. Next, using the Delphi methodology, a panel of neurologists manifested their level of agreement on the different statements using a Likert-type scale. Consensus was reached when ⩾70% of respondents expressed an agreement or disagreement using a five-point scale.

Consensus was achieved on 47 out of 63 recommendations after three rounds of evaluations. The panel considered it essential to address family planning in all patients of childbearing age. There was also consensus that treatment should not be delayed due to the patient’s desire for pregnancy. Additionally, in highly active patients, planning the pregnancy in the medium to long term using depletory drugs such as cladribine or alemtuzumab might represent a useful strategy. However, risks of adverse effects on the fetus due to drug-associated secondary autoimmunity should be addressed when alemtuzumab is considered. Moreover, the maintenance of natalizumab during pregnancy in very active patients reached expert consensus. Also, the panel supported the use of certain disease-modifying treatment (DMT) during lactation in selected cases.

Our results identified specific areas of pregnancy planning in MS patients, where different treatment strategies might be considered to facilitate a safe and successful pregnancy while maintaining clinical and radiological stability.

## Introduction

Multiple sclerosis (MS) is a chronic autoimmune and neurodegenerative disease of the central nervous system that commonly affects women of reproductive age [1]. Currently, MS has no cure, but in the previous decades, an increasing number of Disease Modifying Treatments (DMTs) have been developed for its treatment. The family planning process could be challenging in MS patients of childbearing age, and neurologists need to consider different pharmacological and non-pharmacological issues in order to achieve the best-personalized strategy [2]. This decision-making process can be complex because healthcare providers should balance MS stability in the mother and not interfere with the embryo’s normal development [3]. In order to do so, different risk factors must be taken into consideration to tailor the best medical decision [4]. The major obstetric risk factors mainly include maternal age, co-morbidities, and MS status on the one hand, and pharmacological characteristics of the potentially used DMT on the other [5]. The available DMTs have different mechanisms of action and are associated with specific risks during pregnancy. This particular field underwent several changes in recent years because of the recent authorization of DMT use during pregnancy. Because of this, there has been an increasing number of pregnancies in patients on DMT, and their initial safety reports have shown no significant harm [6,7].

Despite the current existence of international consensus guidelines, there are still many controversial areas about pregnancy in MS. Also, there are uncertainties regarding the best treatment strategy throughout pregnancy and puerperium [8-11]. We aimed to generate an expert consensus addressing relevant recommendations for MS patients on childbearing age concerning pregnancy planning, and antenatal and postpartum care [12], so we carried out the Consenso sobre Planificación en Esclerosis Múltiple Study [Consensus on Planning in Multiple Sclerosis Study] (ConPlanEM Study).

## Materials and methods

A Delphi modified consensus procedure was conducted between 2020 and 2021. Initially, a committee comprising seven neurologists - experts in the MS field - identified the topics to be addressed. Then, a systematic PubMed search was performed in order to prepare evidence-based preliminary statements. Next, three-step Delphi voting rounds were performed, during which a panel of 46 neurologists expressed their level of agreement or disagreement on the different statements using a Likert-type scale. With this scale, the panelist had to specify their level of agreement with each statement using a five-point response key, ranging from 1 (strongly disagree) to 5 (strongly agree). Each consensus round was web-based due to epidemiological concerns because of the COVID-19 pandemic. The first survey used an open-ended round to facilitate feedback from panelists. Subsequent survey rounds focused on topics where consensus was not achieved. Also, statements could be reviewed or modified with new ideas after the first round, according to the panelists’ responses, to facilitate future agreement. Authors and participants based their opinion on local regulations, and changes in safety recommendations may apply in different countries.

Panel selection

The panelists were initially made up of 46 neurologists from 30 Spanish provinces. Of them, a total of 40 completed all the rounds. Panelists were selected as recognized specialists in the clinical and academic fields of MS.

Topic selection and analysis

**Figure 1:**
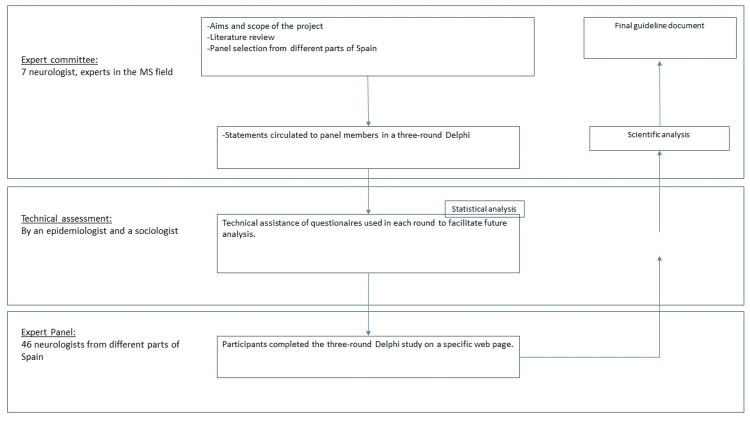
Overview of the workflow used for achieving consensus MS: multiple sclerosis

The consensus was meant to address different topics in the family planning process, including pregnancy planning, antenatal and postpartum care. The clinical scenarios considered included: (1) family planning, contraceptive methods, and fertility assessment, (2) best DMT strategy for family planning, (3) DMT during pregnancy, and (4) treatment strategy after birth. Consensus was achieved if ≥70% of panelists agreed or disagreed on each statement. The workflow of the procedure used in this study is presented in Figure 1.

## Results

As a result, the advisory committee produced 63 real-world recommendations on the proposed main topics (Tables 1-4). The topics included 15 statements related to family planning, 16 concerning DMT before pregnancy, 14 issues associated with MS treatment during pregnancy, and 18 regarding lactation and treatment resumption during postpartum. After the first round of voting, consensus was reached on 26 statements. Next, 19 additional statements underwent a second round of evaluation. After this second round, an agreement was reached on 11 statements. Finally, the remaining statements were revised and underwent an additional round of consensus. After the third round, the panel did not reach a consensus on 16 statements.

**Table 1:** Family planning, contraceptive methods, fertility assessment DMT: disease-modifying treatment, MS: multiple sclerosis, IVF: In vitro fertilization

Statements		Answer (n, % agreement)	Consensus reached at round
Statement 1	Do you consider that it is essential to discuss family planning issues with your patients of childbearing age at MS diagnosis?	Yes (42, 97,6%)	1º
Statement 2	Do you consider that it is essential to discuss family planning with your patients of childbearing age before choosing the first DMT?	Yes (42, 97,6%)	1º
Statement 3	Regarding the use of DMT, do you consider that it should be initiated immediately after MS diagnosis, and it should not be delayed due to a pregnancy desire?	Yes (32, 74,4%)	1º
Statement 4	When considering a DMT election for a female patient, do you think that the childbirth potential should be considered?	Yes (35, 81,4%)	1º
Statement 5	When considering a DMT election for a male patient, do you think that the childbirth potential should be considered?		Not reached
Statement 6	Do you consider it helpful to recommend a fertility assessment when a woman with MS plans to become pregnant, when this choice may have therapeutic consequences?		Not reached
Statement 7	In which of the following clinical circumstances might you recommend postponing pregnancy?		
	a.- clinical activity in the previous year.	Yes 41 (95,3%)	1º
	b.- radiological activity in the previous year.	Yes 33 (76,7%)	1º
	c.- in patients with high disability scores.		Not reached
	d.- in patients with comorbidities		Not reached
Statement 8	Do you think that in vitro fertilization increases the risk of MS relapse?	Yes (36, 83,7%)	1º
Statement 9	Would you contraindicate IVF in a patient with MS?	No (38, 90,4%)	2º
Statement 10	Regarding the risk of inheriting MS, which of the following option do you consider more appropriate?		
	a.-the risk of developing MS increases slightly when only a parent has MS		Not reached
	b.-the risk of inheriting MS is low (2-3%)		Not reached
Statement 11	Would you advise against pregnancy in a patient with MS who has a significant chronic disability?		Not reached

**Table 2:** DMT before pregnancy or best treatment strategy for family planning MS: multiple sclerosis; DMT: disease-modifying treatment; IFN: interferon

Statements		Answer (n, % agreement)	Reached consensus at round
Statement 12	In patients with low-activity MS: should interferons or glatiramer acetate be maintained until the pregnancy is confirmed?	Yes (43,100,00%)	1º
Statement 13	Regarding the use of cladribine in women of childbearing age, which of the following statements do you agree with?		
	a.- Pregnancy should be ruled out before initiation of cladribine in year one and year two.	Agree (38, 88,3%)	1º
	b.- Pregnancy should be avoided by using effective contraception methods during treatment with cladribine and within the following six months.	Agree (42, 97,6%)	1º
	c.- Cladribine would be an ideal drug for family planning in patients of childbearing age.	Agree (39, 90,7%)	1º
	d.- Cladribine would not be an appropriate option in women of childbearing age.		Not reached
Statement 14	Regarding the use of fingolimod, which of the following statements do you agree with?		
	a.- Fingolimod is contraindicated during pregnancy.	Agree (38, 88,3%)	1º
	b.- Patients on fingolimod who are planning a pregnancy need a wash-out period of 2 months, in which the use of contraceptives is mandatory.	Agree (35, 81,4%)	1º
	c.- Considering the risks of rebound after its suspension, it is not an optimal drug when a future pregnancy is being considered.	Agree (33, 76,7%)	1º
Statement 15	In patients on fingolimod who are planning a pregnancy, would you change it to an IFN or glatiramer acetate, considering the possibility of maintaining those treatments throughout the pregnancy to lower the risk of disease rebound after its discontinuation?		Not reached
Statement 16	When planning a pregnancy in a patient on fingolimod, would you change it to another DMT with the safest security profile?	Yes (41, 97,6%)	2º
Statement 17	In patients on natalizumab who manifest pregnancy desire, the benefit/risk of DMT withdrawal should be considered due to the high risk of rebound.	Agree (42, 97,6%)	1º
Statement 18	Considering the different DMT available for MS and considering the risks of disease reactivation after their withdrawal, it is not regarded a good medical practice to leave the patient untreated during the time to pregnancy, especially in cases of highly active disease.	Agree (35, 81,4%)	1º
Statement 19	Would you recommend to a patient to get pregnant six months after the first cycle of cladribine?	Disagree (31, 73,8%)	2º
Statement 20	Would you advise against pregnancy six months after the second cycle of cladribine?	No (28, 70%)	3º
Statement 21	Would you recommend the use of cladribine to a patient with a pregnancy desire in the medium term?	Yes (39, 97,5%)	3º
Statement 22	Would you consider using ocrelizumab in a highly active patient with pregnancy desire in the short term (between 1-2 years)?	No (32, 80%)	3º

**Table 3:** DMT during pregnancy DMT: disease-modifying treatment, IFN-b: Interferon beta, MS: multiple sclerosis, DMF: Dimethyl fumarate

Statements		Answer (n, % agreement)	Reached consensus at round
Statement 23	For patients on IFN-b, it may be considered safe to continue treatment during pregnancy and lactation if it is clinically necessary.	Agree (42,97,6%)	1º
Statement 24	DMF and glatiramer acetate should only be used during pregnancy if the potential benefit justifies the potential risk to the fetus.		Not reached
Statement 25	Would you maintain glatiramer acetate throughout the pregnancy in a treated patient with a controlled disease?	No (29, 72,5%)	3º
Statement 26	In highly active patients, alemtuzumab and cladribine could facilitate family planning, pregnancy, and breastfeeding, due to their pharmacologic characteristics.	Agree (42,97,6%)	1º
Statement 27	In patients on alemtuzumab, the risks of potentially serious adverse effects on the fetus due to drug-associated secondary autoimmunity problems should be considered.	Agree (32, 74,4%)	1º
Statement 28	In highly active MS patients, the possibility of withholding DMT treatment throughout the pregnancy should be considered.	Agree (35, 81,4%)	1º
Statement 29	In case of relapses during pregnancy and its possible treatments:		
	a.- It is recommended to use intravenous methylprednisolone and prednisolone, whereas dexamethasone is contraindicated.	Agree (33, 76,7%)	1º
	b.- The use of plasmapheresis or immunoglobulins may be considered if clinically necessary.	Agree (40, 93%)	1º
	c.- A case of a mild relapse, could not be treated.	Agree (37, 86%)	1º
	d.- If there is a relapse, corticosteroids should be used after the second trimester.		Not reached
	e.- In case of disabling relapses, would you recommend treating it with immunoglobulins?		Not reached
	f.- In case of disabling relapses, would you recommend treating it with plasmapheresis?	Yes 34 (80,1%)	2º
Statement 30	Patients with MS can receive epidural anesthesia and can deliver just like any woman unaffected by MS.	Agree 43, 100%	1º
Statement 31	In a patient with very active MS, would you maintain natalizumab during pregnancy?	Yes (39, 97,5%)	º

**Table 4:** Treatment strategy after birth MS: multiple sclerosis, MRI: Magnetic resonance imaging, DMT: disease-modifying treatment

Statements		Answer (n, % agreement)	Consensus reached at round
Statement 32	To what extent do you trust the protective effect of breastfeeding?	Only mild (34, 88,1%)	2º
Statement 33	In a patient with very active MS, do you consider it appropriate to recommend breastfeeding?	No (32, 76,1%)	2º
Statement 34	In a patient with mild active MS, do you consider it appropriate to recommend breastfeeding?		Not reached
Statement 35	In what circumstances would you promote the initiation of breastfeeding in MS patients?		
	a.- breastfeeding is not recommended if the patient has very active disease.		Not reached
	b.- If desired, exclusive breastfeeding is recommended.		Not reached
	c.- Breastfeeding could be possible in patients on interferon	Agree (34, 79%)	1º
	d.- Breastfeeding could be possible in patients on natalizumab.	Disagree (34,79%)	1º
Statement 36	Do you consider it appropriate to start the following DMT after childbirth and maintain breastfeeding, considering their specific medication leaflet?		
	a.-Cladribine	No (32, 82%)	3º
	b.-Alemtuzumab	No (32, 82%)	3º
Statement 37	When and why would you recommend a follow-up MRI after delivery?		
	a.- Depending on the clinical situation, to help clinical decisions.		Not reached
	b.- If the patient is breastfeeding, MRI should be done during the first trimester of postpartum.	Disagree (39, 90,7%)	1º
	c.- Before the DMT is restarted.	Disagree (36, 83,7%)	1º
	d.- If the patient is a candidate to start high-efficacy DMT, baseline MRI is recommended.	Disagree (34, 79%)	1º
Statement 38	Do you recommend performing an MRI once breastfeeding is completed to select the next DMT according to the inflammatory situation?	Yes (30, 75%)	3º
Statement 39	Would you consider the use of MRI as a decision element to maintain breastfeeding?	Yes (31, 77,5%)	3º
Statement 40	Do you consider it appropriate to initiate and maintain breastfeeding if the patient is in the 3rd and 4th year of cladribine?	Yes (39, 97,5%)	3º
Statement 41	Would you perform MRI in puerperal women who are not willing to breastfeed to adjust for therapeutic reevaluation?	Yes (28, 70%)	3º
Statement 42	Since women with MS may be more susceptible to urinary tract infections, special attention should be paid to detecting and treating these infections.	Agree (37, 86%)	1º

General recommendations

Family Planning, Contraceptive Methods, Fertility Assessment

The panel considered it essential to address family planning in patients of childbearing age, both at the time of disease diagnosis and when the first DMT is being contemplated (Statements 1, 2). Also, there was consensus that the DMT initiation should not be delayed because of the patient’s desire for pregnancy (Statement 3), and the potential risk of future pregnancy should influence the DMT election in female patients (Statement 4).

The panel also agreed that oral contraceptive medications do not significantly interact with DMT (90,7% agreement)[13]. Further, the panel agreed that in vitro fertilization (IVF) increases the risk of MS relapse. Nevertheless, it is not contraindicated when its use is deemed necessary (Statements 8, 9).

In the case of pregnancy desire, the expert panel recommended postponing pregnancy when clinical or radiological activity was present in the previous year (Statement 7 a-b). Conversely, there was no consensus on recommending pregnancy delay in patients with co-morbidities or in those with a significant disability (53.4% did not recommend it, Statement 7 c-d). Also, consensus was not reached on other related topics such as DMT election for a male patient whose partner was planning pregnancy (Statement 5) or the usefulness of future fertility assessment for better pregnancy planning.

Best Treatment Strategy for Family Planning

In this topic, the statements focused on specific treatment strategies for those MS patients who have pregnancy plans, both in the short and long term. All specialists (100%) agreed that interferons and glatiramer acetate in patients with a low-activity MS could be maintained until the pregnancy is confirmed (Statement 12). According to the current evidence, the panel agreed that DMT withdrawal until pregnancy is confirmed should not be considered a good medical practice, especially in highly active patients, due to the potential risk of disease relapse (Statement 18). Nevertheless, participants suggested considering only those DMT options with the best security profile for this situation [14]. Moreover, the panel agreed that cladribine has many pharmacokinetic characteristics that make it a suitable therapeutic option for a patient with active disease, when pregnancy is planned in the medium or long term (Statements 13 and 21), with consensus to recommend conceiving six months after the second year of treatment (Statements 19, 20).

In turn, experts agreed that ocrelizumab should not be recommended at present for highly active patients with a pregnancy desire due to scarcity of data (Statement 22). Also, the panel agreed that as fingolimod is contraindicated during pregnancy, a mandatory washout period is needed, and due to the potential risk of rebound after its withdrawal, this treatment is not a safe choice for pregnancy planning (Statement 14) [15,16]. Because of this, when a patient on fingolimod plans a pregnancy, the panel agreed to change it to another DMT with a safer profile (Statement 16). However, consensus was not reached regarding which option should be chosen in order to reduce the potential risk of disease rebound after the fingolimod withdrawal (Statement 15). Interestingly, consensus was not reached on any particular DMT to be used for this specific situation: 62.5% agreed on the use of glatiramer acetate, 57.5% agreed on cladribine, 57.5% agreed on interferons, and 45% agreed on natalizumab. More importantly, a consensus was reached on which treatment should not be used after fingolimod withdrawal. In this situation the panel specifically did not recommend the use of alemtuzumab (75%), dimethyl fumarate (82,5%), ocrelizumab (87.5%), rituximab (90%), and teriflunomide (100%).

After feedback was received from the initial rounds, the expert committee decided to focus on the panel’s opinion on DMT election for pregnancy planning in two specific clinical situations. First, the panelists were asked which DMT would consider appropriate for short- to medium-term pregnancy planning. The panel agreed that the feasible options were glatiramer acetate (97,5% agreed), cladribine (80% agreed), and interferons (95% agreed). Conversely, neither fingolimod (92,5% agreed), nor ocrelizumab-rituximab (92,5% agreed), nor teriflunomide (92,5% agreed) were recommended for this purpose. Finally, alemtuzumab (62.5% agreed on its use), dimethyl fumarate (60% agreed on its use), and natalizumab (62.5% agreed on its use) did not reach a consensus for this topic. Second, the panelists were asked which DMT they would recommend for pregnancy planning in very active MS patients and which treatment they would prefer in order to reduce the risk of disease reactivation during this period. In both questions, experts agreed to recommend cladribine (92,5%), natalizumab (80%), and alemtuzumab (77.5%). In contrast, the panel agreed on not to recommend the use of glatiramer acetate (77.5%), interferons (70%), teriflunomide (100%), and fingolimod (92%) in this clinical situation. Other treatment options that did not reach consensus were dimethyl fumarate (only 45% agreed on its use) and ocrelizumab (only 32,5% agreed on its use).

DMT During Pregnancy

MS patients should receive obstetric care planning for pregnancy and postpartum like any other woman unaffected by MS (100% agreement) [17]. The experts agreed that interferon beta (IFN-b) might be considered safe during pregnancy and lactation (Statement 23), depending on the patient’s clinical situation. Furthermore, the panel agreed to withdraw glatiramer acetate once the pregnancy is confirmed, in treated patients whose disease is under control (Statement 25). In these lines, consensus was not reached on the use of dimethyl fumarate and glatiramer acetate during pregnancy (Statement 24).

On highly active patients, the panel agreed that maintaining the DMT during pregnancy should be considered (Statement 28). In this line, it was agreed that alemtuzumab and cladribine could be prescribed for pregnancy planning in highly active patients due to their specific pharmacokinetics that allow the patient to be treated for a long time after the drug is eliminated from the organism (74.4% agreement). Nevertheless, the panel also agreed that the risks of potentially serious adverse effects on the fetus due to drug-associated secondary autoimmunity concerns should be addressed when alemtuzumab is considered (Statement 27). Also, the maintenance of natalizumab during pregnancy in very active patients reached expert consensus (97.5% agreement).

Next, the panel was asked about disease reactivation during pregnancy. They agreed that a mild relapse could be left untreated if it has minimal functional implications (86% agreement). When necessary, the panel agreed that relapses could be treated with intravenous methylprednisolone or prednisone (76.7% agreement). In cases of severe relapses, plasmapheresis or intravenous immunoglobulins should be considered (93% agreement) (Statement 29).

Treatment Strategy After Birth

Considering published evidence, experts agreed that breastfeeding only confers a mild protective effect to disease reactivation during postpartum (88.1% agreement) [18]. The authors also agreed to avoid breastfeeding in highly active patients (76.1%), but consensus was not reached on mild-active patients (only 66.6% agreed for lactation). When the mother desires lactation, the panel recommended the use of glatiramer acetate (80.9% agreement) or IFN-b (92.8% agreement) and cladribine in the third and fourth years of treatment (97.5% agreement).

Regarding the indication of a follow-up magnetic resonance imaging (MRI) during postpartum, the panel agreed that an MRI is not essential to initiate breastfeeding (Statement 37), but it could be helpful in the decision to start, maintain, or change a specific DMT according to the radiological activity (Statements 38, 39).

## Discussion

This article provides a comprehensive discussion of issues that may interest MS specialists in assisting patients of childbearing age and family planning. The Delphi process addressed different areas of uncertainty concerning MS care during antenatal and postpartum, including efficacy, safety, and timing of DMT. Overall, a consensus was reached on 47 (74%) statements. In summary, the panel supported that family planning must be considered from disease diagnosis in patients of childbearing age, and it should influence the treatment election. Moreover, the correct selection of the first DMT in a woman of childbearing age could be essential in order to facilitate a future pregnancy process. In this line, a personalized approach is mandatory for each patient, making it difficult to suggest universal recommendations on this issue. Nevertheless, the panel supported the current possibility of using specific DMT during pregnancy and postpartum in selected patients to prevent disease reactivation. Family planning in women with high disease activity was a particular situation where reaching a consensus was more challenging. In selected cases, the panel agreed on using immune reconstitution therapies or natalizumab when pregnancy is planned in patients with high disease activity.

Various autoimmune diseases can lose their clinical stability during pregnancy and postpartum, probably due to the immunological tolerance that the mother’s system generates to their offspring [19-22]. In MS, it was demonstrated that there is a progressive reduction of relapses during pregnancy, becoming infrequent during the third trimester of gestation. In the postpartum period, it was shown that nearly 30% of patients with MS could have a relapse, and different predisposing factors have been identified, such as high relapse rate before pregnancy or high disability status [21,22]. In recent years, it was proposed that the classical improvement of disease activity during the third trimester of pregnancy can be due to the more frequent use of DMTs and their discontinuation at conception [23]. Currently, the best treatment strategy to prevent disease activity fluctuation during this period represents an unmet need [17,24]. The available therapeutic arsenal to treat MS includes drugs with different mechanisms of action, different pharmacokinetics, and different obstetric risk. Some of the DMTs maintain their therapeutic effect only while they are being administered. Other DMTs have a therapeutic impact that exceeds the permanence of the drug in the system. For this reason, the correct DMT election that a childbearing-age woman will receive is essential, and an eventual unplanned pregnancy must also be taken into account. The obtained results regarding the role of a follow-up MRI after delivery in order to adjust decisions supporting breastfeeding, or DMT resumption (Statements 37 and 38) are in line with recent guidelines [25].

Although in vitro fertilization (IVF) was classically associated with a higher risk of MS relapses, especially if patients were treated with gonadotrophin-releasing hormone antagonists, this infertility treatment procedure is not formally contraindicated if patients are not able to conceive naturally. Furthermore, recent studies suggest that in vitro fertilization (IVF) may not be linked with MS relapses, as previously proposed. This improvement in disease control during IVF could be explained, at least in part, by advancements in the treatment landscape for MS and better family planning [26]. In this vein, recent data has shown that continuing the use of disease-modifying therapy (DMT) until receiving a positive pregnancy test after IVF could help reduce the incidence of relapses [27].

The limitations of this study are related mainly to the Delphi research technique itself, which might include panel selection bias and limited possibilities of exchanging opinions and profound discussions on topics that may generate disagreement, given the online nature of the study. However, having selected a large panel of experts from different parts of Spain and considering the opportunity to revise many questions after the received feedback, we believe that the opinions expressed in this document may be representative of different MS specialists. On the other hand, despite the results of our study, the authors pointed out that some discrepancies might exist regarding recommendations between European and other regional guidelines. For example, differences might be seen in the use of anti-CD20 therapies or dimethyl fumarate [9,28]. The authors believe that this different safety recommendation may become obsolete in the near future when further real-world data would be available. Additionally, medical science is in constant evolution. Along this line, the present study was conducted between 2020-2021, before the release of newer DMTs, such as Sphingosine-1-phosphate receptor modulators (i.e., ponesimod) or ofatumumab. Therefore, those treatments were not considered in this work. Nevertheless, newer treatments in MS usually come to market with pregnancy-related safety restrictions, influenced mainly by its pharmacokinetics and pharmacodynamics.

An understanding of the exact mechanisms of action of each DMT and their potential relationship with gestation risk is required when selecting a new treatment for a woman with MS [29]. When a patient is planning a pregnancy, the current medical opinion supports the use of certain DMT during pregnancy if required, as is the case of glatiramer acetate, IFN-beta in patients with low or moderate activity. Conversely, immune reconstitution therapies or natalizumab should be considered for pregnancy planning in patients with high disease activity [30-32]. Some particular options, such as fingolimod or ocrelizumab should be discontinued before pregnancy due to risk concerns [33-35]. Therefore, multidisciplinary management and a risk-benefit evaluation are required for family planning in MS patients.

## Conclusions

In conclusion, the consensus statements presented here aim to assist physicians in various pregnancy and postpartum planning topics in patients with MS. Unfortunately, the data on this field is insufficient, and there is a lack of evidence-based information. The experts agreed that further supporting studies, preferably including data from large cohorts of patients, are strongly required to provide a representative picture of the best treatment planning for women of childbirth age with MS.

## References

[1] Reich DS, Lucchinetti CF, Calabresi PA (2018). Multiple Sclerosis. N Engl J Med.

[2] Oreja-Guevara C (2020). Family planning is the second most relevant factor for treatment decisions after disease activity - No. Mult Scler.

[3] Vukusic S, Michel L, Leguy S, Lebrun-Frenay C (2021). Pregnancy with multiple sclerosis. Rev Neurol (Paris).

[4] Almouzain L, Stevenson F, Chard D, Rahman NA, Hamilton F (2021). Switching treatments in clinically stable relapsing remitting multiple sclerosis patients planning for pregnancy. Mult Scler J Exp Transl Clin.

[5] Krysko KM, Bove R, Dobson R, Jokubaitis V, Hellwig K (2021). Treatment of women with multiple sclerosis planning pregnancy. Curr Treat Options Neurol.

[6] MacDonald SC, McElrath TF, Hernández-Díaz S (2019). Use and safety of disease-modifying therapy in pregnant women with multiple sclerosis. Pharmacoepidemiol Drug Saf.

[7] Thöne J, Thiel S, Gold R, Hellwig K (2017). Treatment of multiple sclerosis during pregnancy - safety considerations. Expert Opin Drug Saf.

[8] Liguori NF, Alonso R, Pinheiro AA (2020). Consensus recommendations for family planning and pregnancy in multiple sclerosis in argentina. Mult Scler Relat Disord.

[9] Dobson R, Dassan P, Roberts M, Giovannoni G, Nelson-Piercy C, Brex PA (2019). UK consensus on pregnancy in multiple sclerosis: 'Association of British Neurologists' guidelines. Pract Neurol.

[10] Batista S, Martins da Silva A, Sá MJ (2020). [Recommendations about multiple sclerosis management during pregnancy, partum and post-partum: consensus position of the Portuguese Multiple Sclerosis Study Group]. Acta Med Port.

[11] Fragoso YD, Adoni T, Brooks JB (2018). Practical evidence-based recommendations for patients with multiple sclerosis who want to have children. Neurol Ther.

[12] Sørensen PS, Centonze D, Giovannoni G (2020). Expert opinion on the use of cladribine tablets in clinical practice. Ther Adv Neurol Disord.

[13] Finkelsztejn A, Brooks JB, Paschoal FM Jr, Fragoso YD (2011). What can we really tell women with multiple sclerosis regarding pregnancy? A systematic review and meta-analysis of the literature. BJOG.

[14] Alroughani R, Altintas A, Al Jumah M (2016). Pregnancy and the use of disease-modifying therapies in patients with multiple sclerosis: benefits versus risks. Mult Scler Int.

[15] Meinl I, Havla J, Hohlfeld R, Kümpfel T (2018). Recurrence of disease activity during pregnancy after cessation of fingolimod in multiple sclerosis. Mult Scler.

[16] Barry B, Erwin AA, Stevens J, Tornatore C (2019). Fingolimod rebound: a review of the clinical experience and management considerations. Neurol Ther.

[17] Langer-Gould AM (2019). Pregnancy and family planning in multiple sclerosis. Continuum (Minneap Minn).

[18] Alhomoud MA, Khan AS, Alhomoud I (2021). The potential preventive effect of pregnancy and breastfeeding on multiple sclerosis. Eur Neurol.

[19] El Miedany Y, Palmer D (2021). Rheumatology-led pregnancy clinic: patient-centred approach. Clin Rheumatol.

[20] Castro-Gutierrez A, Young K, Bermas BL (2021). Pregnancy and management in women with rheumatoid arthritis, systemic lupus erythematosus, and obstetric antiphospholipid syndrome. Med Clin North Am.

[21] Confavreux C, Hutchinson M, Hours MM, Cortinovis-Tourniaire P, Moreau T (1998). Rate of pregnancy-related relapse in multiple sclerosis. Pregnancy in Multiple Sclerosis Group. N Engl J Med.

[22] Vukusic S, Hutchinson M, Hours M, Moreau T, Cortinovis-Tourniaire P, Adeleine P, Confavreux C (2004). Pregnancy and multiple sclerosis (the PRIMS study): clinical predictors of post-partum relapse. Brain.

[23] Alroughani R, Alowayesh MS, Ahmed SF, Behbehani R, Al-Hashel J (2018). Relapse occurrence in women with multiple sclerosis during pregnancy in the new treatment era. Neurology.

[24] Linker RA, Chan A (2019). Navigating choice in multiple sclerosis management. Neurol Res Pract.

[25] Wattjes MP, Ciccarelli O, Reich DS (2021). 2021 MAGNIMS-CMSC-NAIMS consensus recommendations on the use of MRI in patients with multiple sclerosis. Lancet Neurol.

[26] Oreja-Guevara C, Rabanal A, Rodríguez CH, Benito YA, Bilbao MM, Gónzalez-Suarez I, Gómez-Palomares JL (2023). Assisted reproductive techniques in multiple sclerosis: recommendations from an expert panel. Neurol Ther.

[27] Mainguy M, Tillaut H, Degremont A (2022). Assessing the risk of relapse requiring corticosteroids after in vitro fertilization in women with multiple sclerosis. Neurology.

[28] (2023). OCREVUSTM (ocrelizumab) injection. https://www.accessdata.fda.gov/drugsatfda_docs/label/2017/761053lbl.pdf.

[29] Voskuhl R, Momtazee C (2017). Pregnancy: effect on multiple sclerosis, treatment considerations, and breastfeeding. Neurotherapeutics.

[30] Van Der Walt A, Nguyen AL, Jokubaitis V (2019). Family planning, antenatal and post partum care in multiple sclerosis: a review and update. Med J Aust.

[31] Tisovic K, Amezcua L (2019). Women's health: contemporary management of ms in pregnancy and post-partum. Biomedicines.

[32] Kalincik T, Diouf I, Sharmin S (2021). Effect of disease-modifying therapy on disability in relapsing-remitting multiple sclerosis over 15 years. Neurology.

[33] Roy R, Alotaibi AA, Freedman MS (2021). Sphingosine 1-phosphate receptor modulators for multiple sclerosis. CNS Drugs.

[34] He D, Zhang C, Zhao X, Zhang Y, Dai Q, Li Y, Chu L (2016). Teriflunomide for multiple sclerosis. Cochrane Database Syst Rev.

[35] Shah S, Eckstein C (2019). B cell depletion and pregnancy: review and applications for MS treatment. Mult Scler Relat Disord.

